# Availability of brands of six essential medicines in 124 pharmacies in Maharashtra

**DOI:** 10.7189/jogh.08.010402

**Published:** 2018-06

**Authors:** Colin Millard, Abhay B Kadam, Rushikesh Mahajan, Allyson M Pollock, Petra Brhlikova

**Affiliations:** 1 Institute of Health and Society, Newcastle University, Newcastle, United Kingdom; 2 Lakshya, Society for Public Health Education and Research, and Foundation for Research in Community Health, Mumbai, India; 3 Tata Consultancy Services, Mumbai, India

## Abstract

**Background:**

The aim of this study is to assess the availability and rational use of six essential medicines in private retail outlets in Maharashtra state. The study focuses on the range of brands for each medicine, and the availability of these brands in the pharmacies. The medicines were chosen because they are included in the World Health Organization’s (WHO) essential medicines list (EML), the Indian national and Maharashtra state medicines list, and are all included in existing Indian public health initiatives and national disease control programmes.

**Methods:**

Data was gathered on the availability of the medicines and the range and frequency of brands in 124 private retail pharmacies between January and May 2012. As there is currently no centralised database in India of available pharmaceutical brands, we collected data on the range of products of the 6 essential medicines available in the Indian market by consulting three open access Indian pharmaceutical databases, CIMS India, Medindia, and Medguide, and the commercial database, Pharmatrac; we compared this data with the results of the survey. The six essential medicines used in this study are: artemisinin (malaria), lamivudine (HIV/AIDS), rifampicin (tuberculosis control), oxytocin (reproductive health), fluoxetine (mental health) and metformin (diabetes).

**Results:**

The study found that for each of the selected medicines there were multiple approved products listed in Indian databases, 2186 in total. The Pharmatrac database lists only 1359 brands of the selected medicines; 978 (72%) of these had zero sales in 2011-2012. Our survey found very low availability of the brands: 17% Pharmatrac marketed brands (163/978) and 12% of all Pharmatrac brands (163/1359) were available. Metformin was the only medicine with high availability in the study pharmacies at 91%, Rifampacin was the second highest at 64.5%; the other four medicines were available in less than half the pharmacies. A small number of brands were dominating the market.

**Conclusion:**

the survey shows that market competition has generated a large number of brands of the six study medicines but this has not translated into sufficient availability of these medicines in the study pharmacies. The data calls for a review of available brands, taking into consideration levels of sale and grounds for approval, and the setting up of a centralised database of registered pharmaceutical products.

Essential medicines are defined by WHO as medicines which “satisfy the priority health care needs of the population”, they are, “selected with due regard to public health relevance, evidence on efficacy and safety, and comparative cost-effectiveness” [1]. The WHO published its first model list of essential medicine in 1977 (WHO EML) with the purpose of helping governments in low resource settings prioritise their spending on pharmaceuticals [1]. Given that an estimated 70% of pharmaceuticals on the global market are nonessential or duplicative, the aim of a limited list of essential medicines is to enable rational use, lower costs and improve access [2].

Health is a fundamental human right and access to essential medicines is a vital component of a functioning health system. Despite a wave of international initiatives to achieve universal access to safe and effective medicine, enshrined most recently in the Sustainable Development Goal 3.8, it is estimated that at least a third of the global population lacks access to medicines [3]. A recent United Nations report states that essential medicines are available in only 51.8% of public and 68.5% of private health facilities in low and middle income countries [4]. With low availability in the public health sector, patients must turn to the private sector where the prices of generic medicines are 2 to 3.5 times higher than international reference prices [3].

The WHO EML has a strong influence on the medicine policy of many countries. When the list was first published it contained 186 medicines, since that time the number has steadily increased; the list currently contains 374 medicines, and 156 WHO member states have now adopted a medicine list [3]. India established its first National Essential Medicine List (NLEM) in 1996. The list was revised in 2003, 2011 and most recently in 2015 [5]. The 2015 NLEM contains 376 medicines that meet the twin criteria of Indian disease prevalence and cost-effectiveness. Medicines in the NLEM are categorised for their essentiality at Primary (P), Secondary (S) and Tertiary levels (T). The NEML is used as the basis for each Indian state to form its own EML by adding or deleting selected medicines.

Health services in the public sector are provided free of charge and essential medicines listed on the NLEM should be available in public sector outlets but numerous studies have shown inadequate availability [6-8], causing many patients to turn to the private sector. Part of the problem is government funding; World Bank data for 2011 show India’s public health expenditure at 1.2% of GDP, which is amongst the lowest in the world [9]. At the time of independence, private health sector utilisation was only 5%-10% [10]; today out-of-pocket expenditure on private health accounts for 80% of India’s total health expenditure [11]. Patients lose out in that prices of essential generic medicines in the private sector can be 2 to 3.5 times higher than international reference prices [3].

A number of studies have been done on the prices and availability of medicines in India. Four studies used a methodology developed by WHO and Health Action International [12] to measure prices of medicines in low and middle income countries [13-16]. Each of these four studies evaluated the price and availability of medicines in both private and public sector facilities. As patients do not pay for medicines in public facilities, in this sector the studies collected data on procurement prices. All four of the studies showed that in the public sector, although procurement prices were low, availability was poor, while in the private sector, generic drugs were available but at a high price compared with the international reference price. A study of the availability of 27 essential medicines in six Indian states in public sector outlets found low median availability of 0%-30% [6]. A study in Maharashtra State of the availability of 10 essential medicines in 36 primary health centres in 12 districts found insufficient stock of five essential medicines in 75% of the centres and no availability in 13% [17].

The inadequate availability of essential medicines in public health outlets stands in stark contrast to India’s status as a major producer and exporter of generic medicines. There are presently more than 10 500 pharmaceutical manufacturing units in India [18]. Indian companies produce around 60 000 generic drugs and over 400 bulk drugs used in formulations [19]. The industry is ranked third globally in terms of volume and 14th in terms of value, and is growing at 16% per annum. India currently exports off-patent generic drugs to more than 200 countries, for this reason it is often dubbed “the pharmacy of the world” [20]. In theory, competition within India’s vast market for generic drugs should ensure that essential medicines are available in private retail outlets at a price people can afford. The aim of this study is to assess the effectiveness of market competition in contributing to the availability of six essential medicines in private retail outlets in Maharashtra state.

## METHODS

### Study medicines

The present study forms part of an EU-FP7 research project on Accessing Medicine in Africa and South Asia. It focuses on the availability in private retail pharmacies in Maharashtra state, India, of six essential medicines. In India medicines are generally marketed and prescribed by brand name, not generic name. For this reason it was first necessary to assess the range of products within the pharmacies with specific trade names or brand names for each of the six medicines. The range of products available in the pharmacies for each of the medicines was then compared with available data on the full range of brands available for each medicine in India; this data was obtained from three open access databases (CIMS India, Medindia, and Medguide) and one commercial database (Pharmatrac). We included data on the medicines in both single drug formulations (SDF) and fixed dose combinations (FDC).

One of the reasons for choosing Maharashtra as the focus of this study is there is national disease control programmes in operation within the state related to the conditions associated with the six selected medicines [21,22]. Each of the six essential medicines which are the focus of this study are included in the WHO EML, the Indian NLEM and the Maharashtra state EML. Artesunate (P, S, T), a water soluble derivative of artemisinin, is listed in the NLEM for the treatment of malaria. India has a high prevalence of malaria with around 2 million reported cases and 1000 annual deaths [23]. Lamivudine (S,T) is included in the section for antiretroviral medicines for the treatment of HIV/AIDS, in a number of fixed dose combinations. The single drug formulation of lamivudine was deleted from the list in 2015, after this study. According to the the UNAIDS Gap Report [24] India has the third largest HIV epidemic with 2.1 million people living with the illness. Rifampicin (P,S,T), is listed in the NLEM for the treatment of both Tuberculosis and Leprosy. India has the highest levels of tuberculosis (TB) infection in the world. In 2013, India’s 2.1 million TB cases made up almost a quarter of the 9 million global incidence [25]. India also has the highest prevalence of Leprosy with 134 752 new case reported in 2012 [26]. Oxytocin (S,T) is listed in the NEML for reproductive health. It is the internationally established first line drug used for active management of third stage labour. Although the maternal mortality ratio has been progressively declining in India, the estimated figure of 174 for 2015 remains high with 45 000 maternal deaths that year [27]. Metformin (P,S,T) is listed in the NELM for diabetes. The number of people diagnosed with diabetes in India is increasing; there are 69.2 million people with the disease in the country which is the second highest after China’s 109.6 million [28]. Fluoxetine (P,S,T) is included in the NLEM for the treatment of depression. A recent systematic review of the epidemiology of psychiatric disorders in India estimates 20% of the adult population in India to be affected by some form of psychiatric disorder [23]. A study giving an overview of Indian research on depression notes studies vary in their estimates on prevalence in India from 1.7 to 74 per thousand population [29].

### Setting

**Figure 1:**
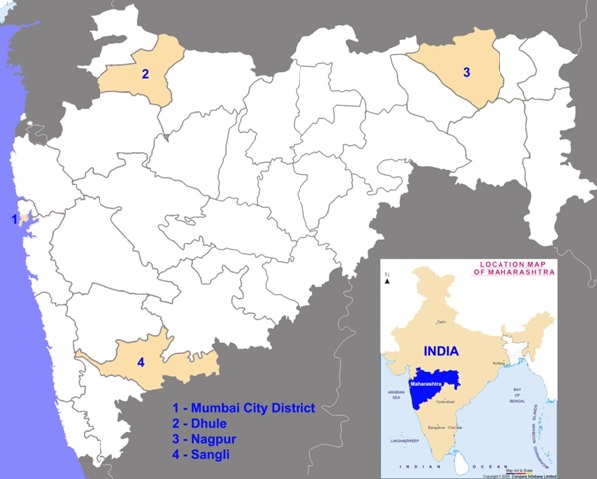
The four study districts in Maharashtra, India.

A further reason why we chose Maharashtra as the location for this research is it has the largest number of manufacturing plants of all Indian states, contributing to 38% of the country’s medicine exports [30]. It also has the largest number of sales units, which the Maharashtra Food and Drug Administration estimates at 80 000 [31]. We determined availability of the medicines in four districts representing each geographical zone of the state: Dhule in the north, Sangli in the south, Nagpur in the east and Mumbai city district in the west (**Figure 1**). These districts were all classed by the Indian government as category A for HIV/AIDS, meaning that more than 1% of women who had been screened at antenatal clinics in the previous three years had the infection. The districts also have high rates of TB and Malaria. **Table 1** gives the TB rate for each district and the percentage of *Plasmodium falciparum* (PF) malaria, the form treated by artemisinin based combination therapy. Retail pharmacies were purposively sampled based on their proximity to primary, secondary and tertiary public health facilities in urban and rural areas. 30 retail pharmacies were selected from Nagpur, 30 from Mumbai, 33 from Dhule and 31 from Sangli districts; similar numbers were selected from urban and rural areas, with the exception of Mumbai which is entirely urban. In the rural areas, from randomly selected tuberculin units, we purposively selected one sub district hospital, one rural hospital and one primary health centre. In the survey, we chose two retail pharmacies located near each of these health facilities. In the urban areas, from randomly selected tuberculin units, we purposively selected one district level hospital, one corporation hospital and one health post. We chose two retail pharmacies located near each of these health facilities. Data was gathered in the local language on the availability of the selected medicines from pharmacists working in the 124 pharmacies in four districts during January to May 2012.

**Table 1:** District pharmacies and disease profiles for 2009-10

Districts	All allopathic pharmacies (public and private) registered under state regulations	Private retail pharmacies under Druggists and Chemists Association (interviewed)	Population (in millions) 2011 Census	TB Rate	Malaria PF percentage
Mumbai City District	Not available	Urban 5000	3.09	217/100 000	21.0
		Interviewed 30			
Dhule	2094	Total 700	2.05	140/100 000	31.0
		Urban 280			
		Rural 420			
		Interviewed 33			
Nagpur	4039	Total 3000	4.65	145/10 000	34.0
		Urban 500			
		Rural 2500			
		Interviewed 30			
Sangli	2225	Total 1700	2.82	114/100 000	23.0
		Urban 300			
		Rural 1400			
		Interviewed 31			

TB – tuberculosis, PF – *Plasmodium falciparum*

### Data analysis

Quantitative data from the survey were entered into a data mask using Epi Info v. 3.5.3 (CDC, Atlanta, GA, USA). The data were used to calculate the percentage availability of the study medicines in the private retail pharmacy outlets, by districts, and by urban and rural areas. We recorded the different available formulations, products and brand names of the six selected essential medicines available in the private retail pharmacies and the level of availability. The results from the pharmacy survey were compared to data on approved products available in these four pharmaceutical databases. There is no centralised database in India of available pharmaceutical brands. We collected data on the different brands of the tracer medicines available in the Indian market by consulting three open access Indian pharmaceutical databases: CIMS India, Medindia, and Medguide. These industry databases are primarily reference sources for doctors, patients and the general public; they are not regularly updated and therefore do not include information on all available pharmaceutical brands in India. We also acquired data from Pharmatrac, a commercial database of Indian national pharmaceutical sales run by the pharmaceutical market research company AIOCD Pharmasoftech AWACS Pvt. Ltd.

### Research ethics

The research proposal was cleared by the Institutional Research Ethics Committee of the Foundation for Research in Community Health in Mumbai and by the ethical review procedures of the University of Edinburgh’s School of Social and Political Science. Written permission to conduct research for this study was obtained from government health officials and medical officers including the Secretary of the Ministry of Health and Family Welfare (Maharashtra). All data were anonymised in a secure database.

## RESULTS

### The availability of the tracer medicines in the study pharmacies

Details concerning the availability of the six study medicines in the four districts are shown in **Table 2**. Of the 124 private retail pharmacies in the four districts, 63.7 percent (n = 79) were urban and 36.3 percent (n = 45) were rural. As the Mumbai City District is entirely urban, the percentage of urban private retail pharmacies is high in the sample. The total availability in the retail pharmacies in the study districts of metformin was very high (91.9%) followed by rifampicin (64.5%). The availability of metformin was high in urban as well as rural areas. The availability of oxytocin (42.7%) and fluoxetine (41.1%) was moderate; there was slightly less availability of artemisinin (33.1%). Lamivudine had the lowest availability (22.6%); it had very low availability in the rural areas (4.4%). Oxytocin was available in less than half the study pharmacies in the rural areas (46.7%), and had slightly lower availability in the urban areas (40.5%). In Dhule and Sangli districts, there was higher availability of artemisinin in the rural areas than in the urban areas, whereas in Nagpur higher levels of availability were found in the urban areas.

**Table 2:** The number and percentage of pharmacies where each tracer medicine was found in the four districts (January – May 2012)

Medicines	Nagpur				Dhule				Sangli				Mumbai			Total					
	**15 Urban**		**15 Rural**		**18 Urban**		**15 Rural**		**16 Urban**		**15 Rural**		**30 Urban**		**Rural**	**79 Urban**		**45 Rural**			**124 Grand total**
	N	%	N	%	N	%	N	%	N	%	N	%	N	%		N	%	N	%		%
Metformin	15	100%	12	80%	16	88.9%	13	86.7%	15	93.8%	13	86.7%	30	100%	NA	76	96.2%	38		84.4%	91.9%
Fluoxetine	9	60%	5	33.3%	4	22.2%	1	6.7%	8	50.0%	2	13.3%	22	73.3%	NA	43	54.4%	8		17.8%	41.4%
Rifampicin	13	86.9%	9	60.0%	10	55.6%	5	33.3%	12	75.0%	6	40.0%	25	83.3%	NA	60	75.9%	20		44.4%	64.5%
Lamivudine	5	33.3%	0	0	2	11.1%	1	6.7%	6	37.5%	1	6.7%	13	43.3%	NA	26	32.9%	2		4.4%	22.6%
Artemisinin	9	60.0%	4	26.7%	7	38.9%	7	46.7%	3	18.8%	4	26.7%	7	23.3%	NA	26	32.9%	15		33.3%	33.1%
Oxytocin	11	73.3%	6	40.0%	6	33.3%	6	40.0%	9	56.8%	9	60.0%	6	20.0%	NA	32	40.5%	21		46.7%	42.7%

### The availability of different products of the selected medicines

The top row of **Table 3** shows the number of brands for each medicine recorded in the pharmacy survey. The next 4 rows give the number of available brands for each of the six tracer medicines listed in three open access databases (CIMS, Medindia, Medguide). Following this is the number of brands listed in Pharmatrac. As can be seen there is some divergence in the numbers of brands for each medicine listed in each database. The databases agreed on general levels of availability: the medicine with the highest number of brands was metformin, this was followed by artemisinin, then rifampicin; there were relatively fewer brands of lamivudine and oxytocin. This pattern reflected the availability of brands in the pharmacy survey.

**Table 3:** Number of brands in industry databases (2012) and numbers of brands found in pharmacies surveyed out in 2012

DRUGS	Oxytocin		Rifampicin		Atemesinin		Lamivudine		Fluoxetine		Metformin	
	**SDF**	**FDC**	**SDF**	**FDC**	**SDF**	**FDC**	**SDF**	**FDC**	**SDF**	**FDC**	**SDF**	**FDC**
Number of brands in pharmacy survey (January – May 2012)	6	0	6	17	14	13	5	4	11	3	25	59
Number of brands in CIMS (2012)	19		227		356		73		111		622	
Number of Brands in Medindia (2012)	23		38		154		56		69		219	
Number of brands in Medguide (2012)	25		528		500		101		243		1110	
Total brands (excluding duplicates)	28	2	125	429	472	78	32	74	169	93	170	514
Number of brands in Pharmatrac database (Nov 2011 – Oct 2012)	18	0	22	236	188	68	9	45	80	34	123	536
Number brands in Pharmatrac with zero sales (Nov 2011 – Oct 2012)	6	0	14	92	61	9	4	8	32	18	29	108

SDF – single drug formulations, FDC – fixed dose combinations

In the databases and the pharmacy survey, with the exception of oxytocin, all the tracer medicines have a high number of FDC brands. The number for lamivudine was more than double its SDFs; for metformin it was almost three times the level. The Pharmatrac data on pharmaceutical sales showed that for each tracer medicine a number of brands had zero sales. Across the six medicine in their SDF and FDC forms, zero sales were recorded for 13% to 63% of the available brands in the single year examined (November 2011 – October 2012). The study found that of the brands that were available in India according to the four databases, only a small proportion were available in the study pharmacies. Metformin reported the highest number of products, followed by artemisinin, and then rifampicin.

### The frequency of products of the selected medicines available in the study pharmacies

Data for the frequency of the availability of products of the six tracer medicines in the private retail pharmacies is shown in **Table 4**. The survey found that only a small number of products had relatively high levels of availability across the study pharmacies. **Table 4** shows the top 3 brands with the highest level of frequency for each medicine, based on the brands with a frequency above 3. For example, as we can see in **Table 3** the two medicines with the largest number of brands in the survey are Metformin (74 brands) and Artemesinin (27) brands, but the vast majority of those brands were available in the 3 or less of the study pharmacies.

**Table 4:** Brands with highest frequency of availability in study pharmacies (January – May 2012)

Study Medicine	Strength	Brand Name	Frequency
**Artemisinin SDFs:**			
1. Arthether	150 mg	AB-Ther	8
2. Artesunate	60 mg	Falcigo	8
3. Arthemether	80 mg	Larither	3
		Larinate, Falcinil, Asunate, Amthar, Mdther, Nomart, Maligon-ART, Artither, Rapither AB, Match, Artifact	Less than 3
**Artemisinin FDCs:**			
1. Arthemether 80 mg, Lumfantrine 480 mg	80 mg	Lumerax	13
		Combither, Combither forte, Rezatrin forte, Arte plus CD, Lumether forte, Lumate-AT, Lumeart, Larinate MF kit, Falcigo SP kit	1
**Fluoxetine SDFs**			
1. Fluoxetine	20 mg	Fludac	49
2. Fluoxetine	20 mg	Flunil	23
		Flutin, Prodac, Platin, Faxtin, Flunat, Flux, Fledore, Flugen, Prodep	3 or less than 3
**Fluoxetine FDCs:**			
1. Fluoxetine 20 mg, Alprazolam 0.25 mg	20 mg	Fluwel	4
		Durian, Oleanz plus	1
**Lamivudine SDF:**			
1. Lamivudine	100 mg	Lamivir HBV	2
2. Lamivudine	150 mg	Lavir	2
		Lamivir, Lamidac	1
**Lamivudine FDCs:**			
1. Lamivudine 150 mg, Stavudine 30 mg	150 mg	Lamistar	2
2. Lamivudine 150 mg, Stavudine 30 mg	150 mg	Duovir	2
3. Lamivudine 300 mg, Zidovidine 300 mg, Efavirenz (kit)	300 mg	Duovir-E	2
		Lamivir S, Triomune	1
**Oxytocin SDFs:**			
1. Oxytocin	5 IU	Pitocin	53
2. Oxytocin	5 IU	Gynotocin	7
3. Oxytocin	5 IU	Syntocinone	5
		Oxytocin, Evatocin, Nitocin	3 or less than 3
**Rifampicin SDFs:**			
1. Rifampicin	450 mg	R-Cin	72
2. Rifampicin	450 mg	Macox	3
		Rifalone, Rifaplus, Famcin, Rimactane	1
**Rifampicin FDCs:**			
1. Rifampicin 450 mg, Isoniazid 300 mg	450 mg	R-Cinex	48
2. Rifampicin 450 mg, Isoniazid 300 mg, Ethambutol 800 mg	450 mg	AKT3	12
3. Rifampicin 450 mg, Isoniazid 300 mg, Ethambutol 800 mg, Pyrazinamide 750 mg	450 mg	AKT4	11
4. Rifampicin 450 mg, Isoniazid 300 mg	450 mg	Rimactazid	11
		Forecox, Rinizide, Akurit 3, Akurit, Montonex forte, AKT2, Monto 2, Macox plus, Monto 3, RHE-FD, Rifa I-6	3 or less than 3
**Metformin SDF:**			
1. Metformin		Glycomet + SR	81
2. Metformin		Glyciphage	73
3. Metformin		Gluformin	21
		Gluconorm	20
		Bigomet	7
		Walaphage	5
		Metlong, Okamet	3 or less than 3
**Metformin FDC:**			
1. Metformin, Glipizide		Glynase MF	28
2. Metformin 500 mg, Glimepiride 2 mg		Glycomet GP2	24
3. Metformin 500 mg, Glimepiride 1 mg		Glycomet GP1	22
		Glyciphage G (all)	19
		Gluconorm (all)	12
		Gemer, Gluformin (all), Metaglez, Glykind M	4-9
		Diabend M, Diabetrol, Duotrol SR, EXEED PG plus, Gemer 2, Gemer P, Glimet, Glimet DS, GLIMI DM PLUS, Glimid M, Glimiprex MF, Glimitide plus, Glimster M, Glimster N1, Glimster PM2, Glimy M, Glipizide M, Glizid M, Gluconorm GT, Gluconorm G, Gluconorm G1, Gluconorm G2, Gluconorm GP1, Gluconorm GP2, Gluconorm 80, Gluconorm P, Gluconorm PG1, Gluconorm PG2, Gluformin G, Gluformin G1, Gluformin GP2, Gluformin MF, Glycheck M, Glycinorm M, Glyciphage P, Glyciphage G1, Glyciphage G2, Glyciphage GP, Glyciphage GP1, Glyciphage GP2, Glyciphage MF, Glyciphage MR, Glyciphage P1, Glyciphage P15, Glyciphage PG, Glyciphage PG1, Glyciphage PG2, Glycomet GP2 forte, Glycomet FP, Glycomet G1, Glycomet MF, Glyconorm G, Glyconorm M, Glycontrol MF, Glymester M, Glymester M1, Glymester M2, Glymet DS, Glymet MR, Glymi M2, Glymin, Glymy M, Glyred M, GMR M1, GMT SR, Metaglez, Metaglez forte, Nuzide M, Okamet M, Pioplus 2, Piopod MF, Pioz MF G, Pioz G2, Pioz MF, Tribet 1, Triglaz	3 or less than 3

SDF – single drug formulations, FDC – fixed dose combinations

## DISCUSSION

The aim of this study is to assess the availability of six essential medicines in private retail outlets in Maharashtra state. The concept of essential medicines was developed to promote rational use, lower cost, and improve access. This study found that despite the multiple brands of selected medicines listed in professional and commercial databases only a small fraction was available in private pharmacies.

As **Table 2** shows, of the six essential medicines surveyed in the 124 pharmacies only metformin had high availability, this was followed by rifampicin at 64.5%, the other medicines where available in less than half the pharmacies studied. Market competition has certainly led to the approval of a very large number of brands of the six study medicines. According to the data we gathered from the three professional databases, the six medicines are represented by 2186 approved products each with different brand names; the commercial database Pharmatrac included 1359 brands with 978 of them marketed in 2011-12. This has not led to high levels of availability in the study pharmacies. The results of this study confirm what the studies mentioned in the introduction found, that in India there remains inadequate access to essential medicines. The strength of this study is that to our knowledge it is the first study of essential medicines in India which compares data on availability in pharmacies with data on pharmaceutical products found in commercial and professional databases.

There are three categories of medicine on the market in India. The vast majority of medicines are generics; these are either “branded medicines” or “branded generics”. In India “branded medicines” are generic drugs manufactured by multinational companies or Indian companies and marketed under their original brand name; these are the most popular drugs in the market. “Branded generics” are bioequivalent to the original product but marketed under another brand name by the same company or any other company [32]. As can be seen from the results of this survey, generic drugs on the Indian market are available in single drug formulations (SDFs) and fixed dose combinations (FDCs). There is also a small number of patented specialised drugs on the market in India such as anti-cancer medicines which are imported from the US and Europe.

Medicine packages in India by law display both the non-proprietary scientific generic name, plus the brand name. However, as previously mentioned, in India medicines are generally marketed and prescribed by brand name, not the scientific generic name. For example, in India, metformin, which is the internationally recognised scientific generic name of the drug, is marketed in the form of different products each having specific brand names (74 of these are listed in **Table 4**). Each product is sold at the Maximum Retail Price which is written on the package; this price is applicable throughout India and varies only slightly according to state level taxation. Each product contains the same medicine, but there is a wide variation in prices between different products and thus cost-effectiveness is not assured.

The results of this survey found that only a few of the approved products listed in the databases were available in the study pharmacies. Furthermore, the frequency of the availability of brands in the study pharmacies showed that only a small number of products were dominating the market; most of the products reported in the survey had frequencies of 3 or less. The reason behind the market dominance of certain products is unknown and more work needs to be done to understand prescribing practices, drug promotional activities including kickbacks, pricing and consumer preferences. A limitation of this research was that as we did not collect data on prices we were not able to explore whether this was a factor in the decision to stock particular brands.

Sales figures show that a large proportion of the 2186 approved products of the six essential medicine are available on the Indian market, but there remain questions about whether this level of market competition leads to wider availability of appropriate medicines and their rational use. For instance a large number of the approved products have zero sales and the reason for this remains unexplained. Across the six medicines in their SDF and FDC forms, zero sales were recorded for 13% to 63% of the available brands in the single year examined (November 2011 to October 2012). There are also questions concerning the safety and effectiveness of the numerous available FDC formulations. In 2007, the Indian regulatory body the Central Drugs Standard Control Organization (CDSCO) banned 294 FDCs which had been approved by state authorities but had never received central authorisation; in 2012 a further 45 FDCs were withdrawn [33,34].

The results of the survey show that pharmacies stocked a limited number of brands with considerable variation within and between pharmacies in the brands stocked. There was very high availability of metformin (91.9%) in all study districts and in both urban and rural areas. The national disease control program for diabetes has not been rolled out in all Indian states. In Maharashtra the program is still in its early stages. Metformin is supposed to be available at primary health centres, but this was generally not the case during the period of our study; it was available only in a few public health facilities in Mumbai City District. Diabetes, as a lifelong chronic disorder, provides an assured market for pharmaceutical companies. The high levels of availability in the private retail pharmacies in the four study districts may be accounted for by the poor availability of metformin in public health facilities and the low level of development of the national disease control program in Maharashtra. It may also be the case that metformin has been strategically marketed in the area taking advantage of the low level of public provision.

There was high availability of rifampicin (64.5%) in all study districts with higher availability in urban compared to rural areas. Although the well-established national disease control program for tuberculosis has achieved significant progress over the last decade (RNTCP Status report 2009), studies have reported that 50%-80% of TB patients in India take treatment from private health care facilities [35,36]. Additionally the consumption of first line anti tuberculosis drugs in the private sector market is very high (65%) compared to the use in public health facilities [37].

The study found moderate levels of availability of oxytocin (42.7%) in the study pharmacies, slightly more in the rural areas than in the urban ones. There is a well-developed maternal child health program in India and through this oxytocin is widely available at primary public health facilities, but unqualified staff and poor management of stocks and inadequate storage conditions all contribute towards poor availability of oxytocin in public health facilities and this may account for its availability in the private pharmacies [38-42]. Additionally there is evidence that its availability in private pharmacies has contributed to its misuse to induce or speed labours [38-41].

The study found moderate availability of fluoxetine (41.1%) in the study pharmacies in urban areas and poor levels in rural areas. The national disease control programs for depression and other mental health disorders are not well developed in India. Fluoxetine is included in the Indian national EML but it has low levels of availability in public health facilities. In addition there is a general lack of qualified doctors for prescribing such medicines in primary health care facilities. The low levels of availability in the study pharmacies may also be accounted for by the fact that private sector health facilities for treating depression and other mental health disorders are concentrated at the district headquarters. Other new medicines in the selective serotonin reuptake inhibitors group are now also available in India; this could also be a factor contributing to the low availability of fluoxetine.

With the exception of the urban area of Nagpur, the study showed low availability of artemisinin (33.1%) in both urban and rural private outlets. The national disease control program for malaria is generally well developed in India. However, this was not the case in urban areas of Nagpur district, which would account for the relatively high levels of artemisinin found here in the study pharmacies.

Of all the tracer medicines, lamivudine had the lowest availability in the study pharmacies. The situation was worse in the rural areas where in both Sangli and Dhule only one of the 15 surveyed pharmacies stocked it, and in Nagpur it was not available. The national disease control program for HIV/AIDS in India is relatively new, but it is a high priority program with good levels of funding, and consequently a strong well-staffed infrastructure is now in place. This has improved the availability of anti-retroviral medicines in secondary and tertiary public health facilities in India [43]. Lamivudine is an expensive lifelong treatment and as such the first recourse for people with HIV/AIDS in India is to use free anti-retroviral medicines provided by the national disease control program. This would account for the low availability of lamivudine in the study pharmacies. However, stigma associated with HIV/AIDS remains a problem in India, and this is a major reason why some patients still opt for treatment in private health facilities.

## CONCLUSION

In conclusion, this survey shows that the large number of brands of the six study medicines registered in India, as documented by professional and commercial databases, has not translated into sufficient availability of these medicines in the study pharmacies. A solution would be to strengthen the national disease control programs for conditions associated with the six selected medicines, as they are the major source of free medicines for the large section of the population with limited financial means. In a few instances, this can be attributed to well-functioning public health programs that are a major source of free medicines for the large section of the population with limited financial means. Further strengthening of the national disease control programs for conditions associated with the six selected medicines is necessary as the private sector provision does not sufficiently ensure availability and affordability of medicines.

The present market based system also leads to irrational medicine use. The widespread use of brand names for prescribing is a case in point. It is acknowledged by the Indian Medical Council that the common practice of prescribing medicines by brand names can lead to confusion. In January 2013 it therefore asked doctors to prescribe drugs by scientific generic names to ensure compliance to clause 1.5 of the Indian Medical Council (Professional Conduct, Etiquette and Ethics) 2002 Regulations, which contains the provision that every physician should, as far as possible, prescribe drugs with scientific generic names in order to achieve rational prescribing and use of drugs [44]. This would be in accordance with the WHO’s guidance on the use of international non-proprietary names for safe prescription and dispensing of medicines [45]. The issue of the irrational use of FDCs in India has been addressed by McGettigan et al. [33].

In India there is no central database of products approved for manufacture and marketing by State Licensing Authorities. Recently the union health ministry's expert panel has been constituted in a massive exercise to examine and regularise the thousands of FDCs products (over 5000) permitted to manufacture and sale in the country by states without due approval from the Drugs Controller General of India (Ramesh Shankar, June 2014). CDSCO needs to review the number of brands on the market, taking into consideration sales, availability, price and the grounds for approval. There is also an urgent need for a consumer friendly central database which will enable people to verify manufacturing approval, efficacy and compare prices.
